# Time to Treatment and Patient Outcomes among TB Suspects Screened by a Single Point-of-Care Xpert MTB/RIF at a Primary Care Clinic in Johannesburg, South Africa

**DOI:** 10.1371/journal.pone.0065421

**Published:** 2013-06-06

**Authors:** Colleen F. Hanrahan, Katerina Selibas, Christopher B. Deery, Heather Dansey, Kate Clouse, Jean Bassett, Lesley Scott, Wendy Stevens, Ian Sanne, Annelies Van Rie

**Affiliations:** 1 University of North Carolina Gillings School of Global Public Health, Chapel Hill, North Carolina, United States of America; 2 Clinical HIV Research Unit, Department of Medicine, University of the Witwatersrand, Johannesburg, South Africa; 3 Witkoppen Health and Welfare Centre, Johannesburg, South Africa; 4 Department of Molecular Medicine and Hematology, University of the Witwatersrand, Johannesburg, South Africa; 5 National Health Laboratory Services, Johannesburg, South Africa; University of Cape Town, South Africa

## Abstract

**Introduction:**

In December 2010, the World Health Organization recommended a single Xpert MTB/RIF assay as the initial diagnostic in people suspected of HIV-associated or drug resistant tuberculosis. Few data are available on the impact of this recommendation on patient outcomes. We describe the diagnostic follow-up, clinical characteristics and outcomes of a cohort of tuberculosis suspects screened using a single point-of-care Xpert.

**Methods:**

Consecutive tuberculosis suspects at a primary care clinic in Johannesburg, South Africa were assessed for tuberculosis using point-of-care Xpert. Sputum smear microscopy and liquid culture were performed as reference standards. Xpert-negatives were evaluated clinically, and further assessed at the discretion of clinicians. Participants were followed for six months.

**Results:**

From July-September 2011, 641 tuberculosis suspects were enrolled, of whom 69% were HIV-infected. Eight percent were positive by a single Xpert. Among 116 individuals diagnosed with TB, 66 (57%) were Xpert negative, of which 44 (67%) were empirical or radiological diagnoses and 22 (33%) were Xpert negative/culture-positive. The median time to tuberculosis treatment was 0 days (IQR: 0–0) for Xpert positives, 14 days (IQR: 5–35) for those diagnosed empirically, 14 days (IQR: 7–29) for radiological diagnoses, and 144 days (IQR: 28–180) for culture positives. Xpert negative tuberculosis cases were clinically similar to Xpert positives, including HIV status and CD4 count, and had similar treatment outcomes including mortality and time to antiretroviral treatment initiation.

**Conclusions:**

In a high HIV-burden setting, a single Xpert identified less than half of those started on tuberculosis treatment, highlighting the complexity of TB diagnosis even in the Xpert era. Xpert at point-of-care resulted in same day treatment initiation in Xpert-positives, but had no impact on tuberculosis treatment outcomes or mortality.

## Introduction

Access to rapid and accurate diagnosis of tuberculosis (TB) remains a barrier to TB control–globally only 65% of TB cases were diagnosed in 2011 [1]. The Xpert MTB/RIF assay (Xpert) represents a potential revolution in TB diagnosis, with good accuracy, rapid time to results and the possibility of decentralized implementation close to the point-of-care [2]–[4]. The World Health Organization has endorsed the use of Xpert as the initial diagnostic in individuals suspected of HIV-associated TB or MDR-TB, or as a follow-on test to sputum smear microscopy [5].

South Africa has committed to a national scale-up of Xpert, with the ultimate goal of screening all adult pulmonary TB suspects with a single Xpert performed at a centralized laboratories [6]. Implementing Xpert at the point-of-care has been demonstrated to be safe in terms of infection control [7], and has the potential for same day treatment initiation [8], though may be more expensive than placement in a central laboratory [9]. Moving the test away from the point-of-care could result in delays to results that decrease the potential patient-level impact of Xpert [10]. Few data are available on the impact of Xpert at point-of-care on time to treatment and patient outcomes. Furthermore, although Xpert has a higher sensitivity than smear microscopy [3], [11], [12], reliance on a single Xpert may result in a substantial number of missed TB cases. The few studies presenting diagnostic information on Xpert negative individuals suggest that smear microscopy and chest x-ray have limited utility in detecting culture positive, Xpert negative TB [13], [14].

Using operational data collected during the course of routine clinical care at a primary care clinic in South Africa, we sought to describe the diagnostic follow-up, clinical characteristics and outcomes of a cohort of TB suspects screened using a single point-of-care Xpert.

## Methods

### Study Design

Witkoppen Health and Welfare Centre (WHWC) is a high-volume primary health clinic primarily serving informal settlement communities in northern Johannesburg, South Africa. Since 2011, all patients presenting at the clinic were routinely tested for HIV and screened for the presence of TB symptoms. Consecutive TB suspects who consented were enrolled into this prospective cohort study. As part of the WHWC routine diagnostic process, all TB suspects also were assessed by fluorescent sputum smear microscopy for acid-fast bacilli and liquid culture for *M. tuberculosis*, performed at a centralized National Health Laboratory Service laboratory. In addition, Xpert was performed as a research procedure at point-of-care by a low-skilled health care worker. All results, including Xpert, were used for clinical decision making.

Patients with a TB diagnosis received TB treatment at WHWC, and TB/HIV co-infected individuals were eligible for antiretroviral treatment (ART), while among those without TB co-infection the ART treatment threshold was ≤350 cells/mm^3^. As part of routine care and independent of study participation, Xpert negative TB suspects were treated with a course of antibiotics if clinically indicated, and asked to return for follow-up after one week. At follow-up, Xpert negative TB suspects were evaluated clinically, and had additional diagnostic testing (chest xray and a second Xpert) at the discretion of the clinician. Chest x-rays were performed at a nearby clinic. Chest x-ray results were classified as positive when the radiologist’s report stated the x-ray was suggestive of TB.

Demographic and clinical characteristics as well as laboratory findings were collected through chart review. The clinical status of participating Xpert negative TB suspects was determined through chart review and/or telephone calls at two months from the initial Xpert test, and again at six months for those who remained symptomatic or could not be contacted at two months. Patients lost to follow-up were traced in the national death registry. Treatment outcomes at six months were determined for all those initiated on TB treatment.

### Definitions

The TB suspect definition was stratified by HIV status. For HIV-infected or unknown status: cough, fever, night sweats, weight loss of any duration; for HIV-uninfected: cough or fever ≥ two weeks, or night sweats or weight loss of any duration.

The basis for the decision to initiate TB treatment was defined as the earliest positive diagnostic test (smear, culture, Xpert or xray), or an empiric diagnosis if treatment was started in absence of or prior to any positive diagnostic test.

TB suspects were classified as *confirmed TB* if smear microscopy, culture or Xpert were positive, independent of whether TB treatment was initiated. TB suspects were classified as *possible TB* if started on treatment in the absence of mycobacteriological confirmation (i.e. Xpert negative, smear negative or missing and culture negative, missing or contaminated). TB suspects were classified as *not TB* if culture, smear and Xpert were negative and TB treatment was not started, or if the clinical outcome was good in the absence of TB treatment among those with missing or contaminated culture results.

Time to treatment was defined as the time from the baseline visit, when the initial Xpert test was performed, until initiation of TB treatment, whether at WHWC or elsewhere.

### Statistical Analysis

Standard descriptive statistics were used to characterize the cohort. Multinomial logistic regression was used to estimate odds ratios and their 95% confidence intervals (CI) for the association between baseline characteristics and the Xpert result among TB cases (both definite and possible), using those -classified as not TB as the referent population. Predictors with significance p<0.10 in the univariate analysis were included in the multivariate model. Poisson regression with robust error variance was used to compare characteristics among Xpert positive and negative TB cases, as well as among confirmed and possible TB cases [15]. Time to treatment was compared by basis of TB treatment initiation using Kaplan-Meier curves and the log-rank test. All statistical analyses were conducted using Stata 12 (StataCorp, College Station, TX).

### Ethics Statement

This study was approved by institutional review boards at the University of North Carolina, Chapel Hill and the University of the Witwatersrand in Johannesburg, South Africa. All participants gave written consent for use of routine clinical data for research purposes.

## Results

### Baseline Cohort Characteristics

Between July 15 and September 22, 2011, 641 consecutive TB suspects were consented and enrolled into the study. The median age was 35 years (IQR: 29–44), 65% were female, and 35% were of non-South African nationality, with the majority (73%) from Zimbabwe (see Table 1). Sixty-nine percent were HIV-infected, of whom 45% were on ART at the baseline visit for a median of 21 months (IQR: 12–35). Among those not on ART, the majority (174/280, 62%) were eligible for treatment, with a CD4 count of ≤350 cells/mm^3^. The most common presenting TB symptom was cough (97%), followed by night sweats (34%).

**Table 1:** Characteristics of 641 TB suspects screened by Xpert MTB/RIF assay as the initial TB diagnostic, stratified by baseline Xpert result.

	TB Suspects			
	Initial	Initial		All
Characteristic	Xpert positive(n = 50)	Xpert negative(n = 591)	p value	(n = 641)
Age, median (IQR)	34 (29–39)	36 (29–44)	0.108	35 years (29–44)
Female gender (%)	23(46)	392 (66)	0.004	415 (65)
Non-South African nationality (%)	19 (38)	260 (44)	0.412	223 (35)
History of TB (%)	5 (10)	57 (10)	0.935	62 (10)
Presenting TB symptom				
Cough (%)	49 (98)	569 (96)	0.529	618 (96)
Loss of weight(%)	30 (60)	188 (32)	<0.001	218 (34)
Fever (%)	5 (10)	38 (6)	0.333	43 (7)
Night sweats(%)	33 (66)	181 (31)	<0.001	214 (33)
HIV status			0.026	
Positive	43 (86)	400 (68)		443 (69)
Negative	6 (12)	156 (26)		162 (25)
Unknown	1 (2)	35 (6)		36 (6)
Among HIV positive				
CD4 count, mean (95% CI)	179 cells/mm^3^ (128–230)	344 cells/mm^3^ (319–370)	<0.001	329 cells/mm^3^ (305–353)
CD4 count, median (IQR)	148 cells/mm^3^ (66–249)	288 cells/mm^3^ (151–487)	<0.001	276 cells/mm^3^ (138–458)
CD4 count category			0.001	
CD4≤200 cells/mm^3^ (%)	24 (56)	126 (32)		152 (34)
CD4 201–350 cells/mm^3^ (%)	10 (20)	99 (25)		109 (25)
CD4 351–500 cells/mm^3^ (%)	3 (6)	71 (18)		74 (17)
CD4>500 cells/mm^3^ (%)	2 (4)	92 (23		94 (21)
CD4 missing (%)	4(9)	10 (3)		14 (3)
On ART at first suspect visit (%)	4 (9)	198 (45)	<0.001	202 (45)
Time on ART, median (IQR)	23 months	21 months (11–35)	0.606	21 months
	(14–25)	(11–35)		(12–35)

**Abbreviations:** TB, tuberculosis; IQR, interquartile range; CI, confidence interval; ART, antiretroviral therapy.

### Diagnostic Follow-up and TB Diagnosis

**Figure 1:**
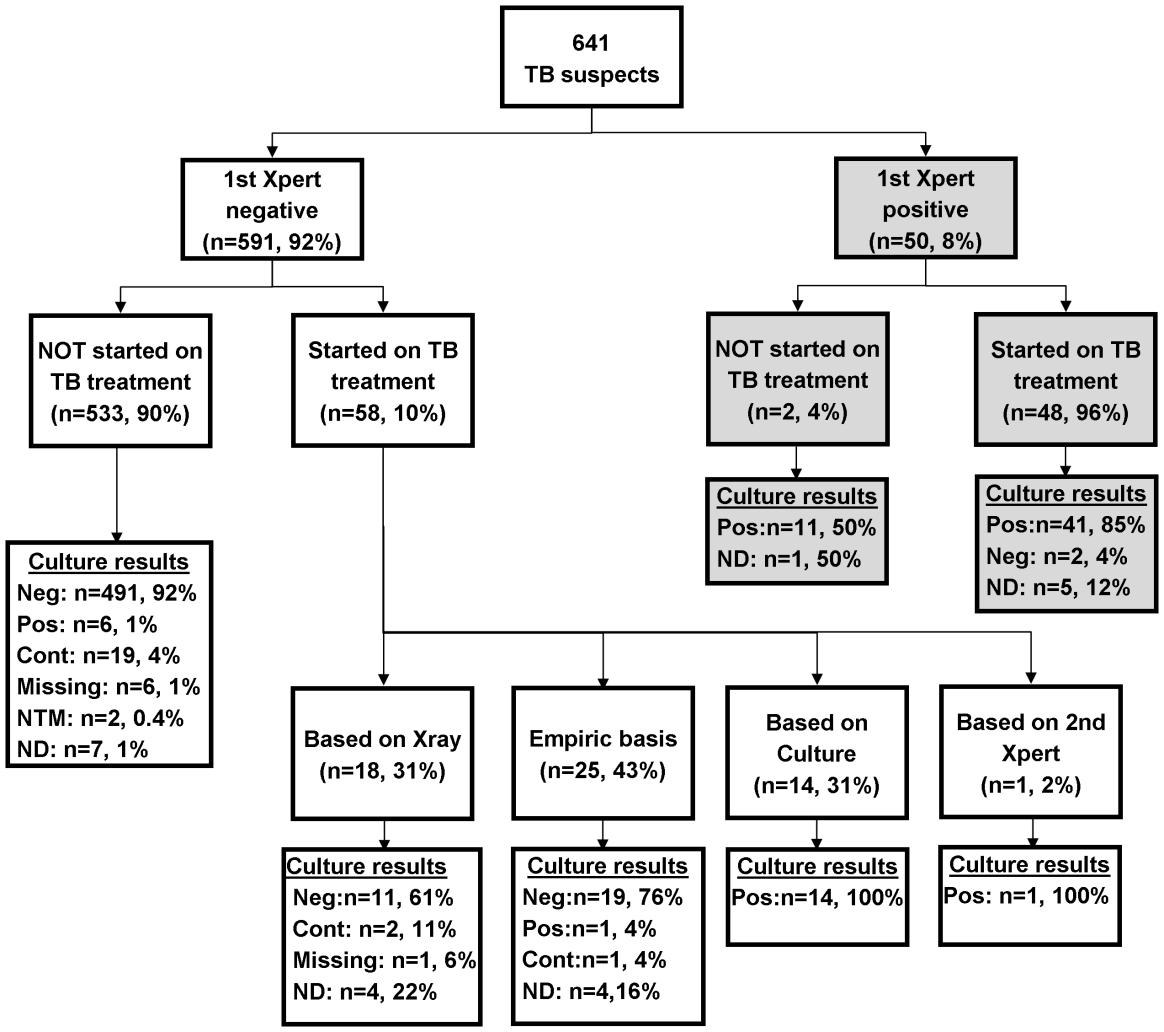
Flow chart of diagnostic assessment and basis of TB treatment initiation in 641 TB suspects presenting to a primary care clinic in Johannesburg, South Africa. The basis of diagnosis was defined as the earliest positive diagnostic test (smear, culture, Xpert or x-ray), or an empiric diagnosis if treatment was started in absence of or prior to any positive diagnostic test. Abbreviation: TB, tuberculosis; Neg, negative; Pos, positive; Cont, contaminated; NTM, non-tuberculous mycobacteria; ND, not done.

The initial Xpert was positive in 8% (50/641) of TB suspects (Figure 1). Among these, 32% (16/50) had a positive smear and culture, 52% (26/50) were smear-negative/culture-positive, 4% (2/50) were culture-negative and 12% (6/50) had missing or unreported laboratory results. Among the 551 TB suspects with a valid culture result, the sensitivity of a single Xpert compared to culture was 66% (95% CI: 53–77%) and the specificity was 99% (95% CI: 98–100%).

The initial Xpert was negative in 92% of suspects (591/641). Among these, 72% were prescribed antibiotic treatment at the baseline visit, with 66% receiving amoxicillin, 17% erythromycin, 11% amoxicillin/clavulanate potassium, and 6% another antibiotic. Among Xpert negative TB suspects, smear microscopy and liquid culture were performed in the majority (98%) of patients, chest x-ray in 15% and a second Xpert in 8%. Culture was positive in 4% of Xpert-negatives (22/591), while smear microscopy was positive in only 1/591 (0.2%). Chest x-ray was performed in 84 and suggestive for TB in 26 (31%), and a second Xpert was requested in 49 and positive in 2. In addition, non-tuberculous mycobacteria grew in the sputum cultures of 2 (4%) Xpert negative TB suspects.

Overall, 11% (66/591) Xpert-negative TB suspects were classified as TB cases, of which 35% were confirmed cases (22 culture-positive, 1 smear-positive culture-negative and 65% possible TB cases (18 radiological diagnosis and 25 clinical diagnosis).

### Factors Associated with TB Diagnosis

Among 641 TB suspects, the odds of receiving a TB diagnosis was higher if HIV-infected (aOR 7.22 [95% CI: 2.71–19.22] for Xpert-positives and aOR 18.96 [95% CI: 6.42–55.98] for Xpert-negatives), or ART-naïve (aOR 5.6 [95% CI: 1.78–17.24] for Xpert-positives and aOR 2.70 [95% CI: 1.43–5.55] for Xpert-negatives) and (Table 2). The presence of night sweats (aOR 2.61 [95%I 1.28–5.30] and loss of weight (aOR 2.29 [1.16–4.52] was associated with having a positive Xpert, while night sweats was the only significant presenting symptom associated with an Xpert-negative TB diagnosis (aOR 3.01 [95% CI: 1.67–5.41]). A higher CD4 count was associated with a lower odds of a TB diagnosis, but this was only significant among Xpert-negative TB suspects (aOR 0.14 to 0.37).

**Table 2:** Multinomial logistic models comparing baseline characteristics between 50 Xpert positive and 66 Xpert negative TB cases compared to those classified as not having TB.

	Univariate							Multivariate			
Characteristic	No TB (REF) n = 525	Xpert-positive TB diagnosis n = 50			Xpert-negative TB diagnosis n = 66			Xpert-positive TB diagnosis n = 50		Xpert-negative TB diagnosis n = 66	
	n (%)	n (%)	OR	95% CI	n (%)	OR	95% CI	aOR	95% CI	aOR	95% CI
**SA nationality**	291(55)	31(62)	REF		40(61)	REF					
**Non SA nationality**	234(44)	19(38)	0.76	0.42–1.39	26(39)	0.81	0.48–1.36				
**No history of TB**	476(90)	45(90)	REF		58(88)	REF					
**History of TB**	49(9)	5(10)	1.08	0.412.84	8(12)	1.34	0.60–2.97				
**Age ≤35 years**	263(50)	31(62)	REF		27(41)	REF		REF		REF	
**Age >35 years**	262(49)	19(38)	0.62	0.34–1.11	39(59)	1.45	0.86–2.44	0.61	0.30–1.24	1.65	0.92–2.94
**Female gender**	354(67)	23(46)	REF		38(58)	REF		REF		REF	
**Male gender**	226(35)	27(54)	2.43	1.35–4.36	28(42)	1.53	0.91–2.57	1.63	0.82–3.29	1.04	0.57–1.89
**HIV status**											
** Negative**	152(29)	6(12)	REF		4(6)	REF		REF		REF	
** Positive**	341(65)	59(89)	3.19	1.33–7.67	43(86)	6.57	2.35–18.4	7.22	2.71–19.22	18.96	6.42–55.98
**Unknown**	32(6)	1(2)	0.79	0.092–6.80	3(5)	3.56	0.76–16.70	0.59	0.067–5.26	0.137	0.68–15.97
**CD4 count**											
** CD4<100**	41(7)	17(34)	REF		25(38)	REF		REF		REF	
** CD4 101–200**	50(9)	8(16)	0.41	0.19–0.91	13(20)	0.33	0.16–0.67	0.78	0.32–1.89	0.37	0.17–0.82
** CD4 201–350**	88(16)	10(20)	0.17	0.048–0.57	12(18)	0.18	0.068–0.49	0.39	0.11–1.48	0.29	0.10–0.82
** CD4>350**	154(29)	10(15)	0.082	0.019–0.36	5(10)	0.14	0.051–0.36	0.28	0.061–1.32	0.14	0.051–0.36
**No ART**	156(45)	39(91)	REF		47(80)	REF		REF		REF	
**ART**	185(54)	4(9)	0.10	0.06–0.29	12(20)	0.24	0.12–0.47	0.18	0.058–0.56	0.37	0.18–0.79
**TB Symptoms**											
** No night sweats**	382(72)	17(34)	REF		28(42)	REF		REF		REF	
** Night sweats**	143(27)	33(66)	5.19	2.80–9.60	38(58)	3.63	2.15–6.13	2.61	1.28–5.30	3.01	1.67–5.41
** No loss of weight**	365(69)	20(40)	REF		38(58)	REF		REF		REF	
** Loss of weight**	35(6)	30(60)	3.42	1.89–6.21	28(42)	1.68	0.99–2.83	2.29	1.16–4.52	1.29	0.72–2.31
** Cough**	507(96)	49(98)	REF		62(94)	REF					
** No cough**	18(3)	1(2)	1.74	0.23–13.31	4(6)	0.55	0.18–1.68				
** No fever**	493(93)	45(90)	REF		60(91)	REF					
** Fever**	32(6)	5(10)	1.71	0.64–4.61	6(9)	1.54	0.62–3.84				

**Abbreviations:** TB, tuberculosis; OR, odds ratio; aOR, adjusted odds ratio; CI, confidence interval; ART, antiretroviral treatment; REF, reference category; SA, South African.

Results in bold are significant at the p<0.05 level.

Among the 116 TB cases, age was the only factor associated with a positive Xpert result, with those over 35 years being 40% less likely to have Xpert positive TB compared to Xpert negative TB (aRR 0.60 [95% CI: 0.36–0.98]) (Table 3). Compared to those with confirmed TB, possible TB s were more likely to be HIV-infected (98% vs 82%, p 0.028), but were similar on other clinical characteristics, including demographics, CD4 count, HAART use and TB symptoms in univariate and multivariate regression analysis (results not shown).

**Table 3:** Poisson regression models with robust error comparing characteristics among 50 Xpert positive and 66 negative TB diagnoses.

	Univariate		Multivariate	
Characteristic	RR (95% CI)	p value	aRR (95% CI)	p value
**SA nationality**	REF			
**Non SA nationality**	1.03 (0.67–1.60)	0.880		
**No history of TB**	REF			
**History of TB**	0.88 (0.43–1.82)	0.730		
**Age ≤35 years**	REF		REF	
**Age >35 years**	0.61 (0.39–0.95)	0.030	0.60 (0.36–0.98)	0.042
**Female gender**	REF		REF	
**Male gender**	1.30 (0.85–1.99)	0.200	1.28 (0.81–2.02)	0.290
**HIV status**				
** Negative**	REF		REF	
** Positive**	0.70 (0.40–1.23)	0.214	0.68 (0.39–1.17)	0.164
** Unknown**	0.42 (0.07–2.47)	0.335		
**CD4 count**				
** CD4<200**	REF		REF	
** CD4≥200**	1.06 (0.66–1.72)	0.807	1.32 (0.49–3.51)	0.583
**No ART**	REF			
**ART**	0.57 (0.23–1.380	0.211		
**TB Symptoms**				
** No loss of weight**	REF		REF	
** Loss of weight**	1.50 (0.97–2.32)	0.068	1.25 (0.79–2.01)	0.337
** No night sweats**	REF			
** Night sweats**	1.23 (0.78–1.93)	0.369		
** Cough**	REF			
** No cough**	0.45 (0.08–2.67)	0.381		
** No fever**	REF			
** Fever**	1.06 (0.53–2.11)	0.867		

Abbreviations: TB, tuberculosis; ART, antiretroviral treatment; RR, relative risk; aRR, adjusted relative risk; REF, reference.

Among culture-positive TB cases, individuals testing Xpert-negative had significantly longer time to culture positivity compared to Xpert-positives (median of 23 days [IQR: 18–32] versus 12 days [IQR: 10–16], p<0.001).

### TB Treatment Initiation

**Figure 2:**
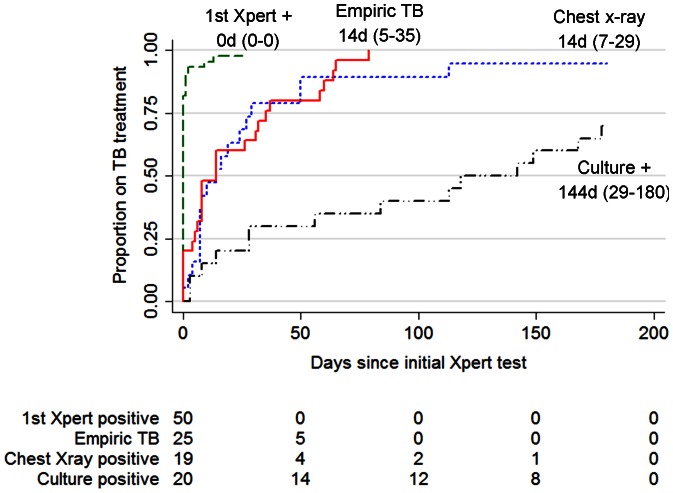
Time to TB treatment in 114 TB cases, by basis of TB treatment initiation. Kaplan-Meier curves showing time to treatment stratified by basis of TB diagnosis, excluding those diagnosed based on 2^nd^ Xpert or sputum smear microscopy (n = 2). The median time to treatment and IQR for each basis of TB treatment initiation are listed. Abbreviation: TB, tuberculosis.

In total, 106 of the 116 TB caseswere started on TB treatment by six months following the initial Xpert assay. Almost all (48/50, 96%) Xpert positive patients started treatment, 2 were lost to follow-up., The proportion of Xpert-negative TB cases starting TB treatment was 70% (14/20) of culture positives, 95% (18/19) of individuals with suggestive chest x-ray, 100% (1/1) with positive 2^nd^ Xpert, and 0% (0/1) with positive smear microscopy. In addition, 25 individuals started empiric TB treatment. The median time to TB treatment was 0 days (IQR: 0–0) for those positive on the initial Xpert, 14 days (IQR: 5–35) for empiric TB, 14 days (IQR: 7–29) for those starting treatment based on suggestive chest x-ray findings, and 144 days (IQR: 28–180) for culture positive, Xpert-negative patients (see Figure 2).

### Two and Six Month Follow-up

**Figure 3:**
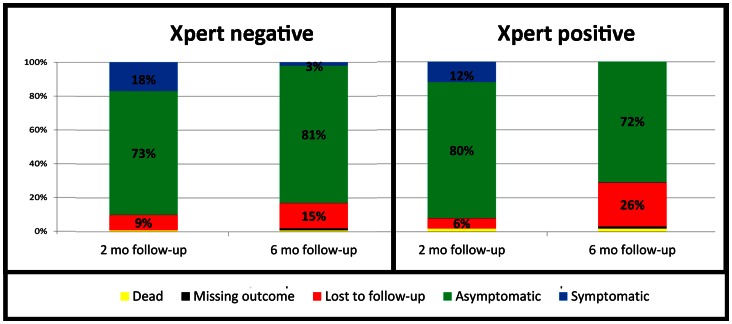
Two and six-month outcomes of 591Xpert-negative TB suspects and 50 Xpert-positive TB suspects presenting to a primary care clinic in Johannesburg, South Africa. Abbreviations: LTFU, lost to follow-up; TB, tuberculosis; mo, month; Rx, treatment.

Among TB suspects with a negative initial Xpert, 18% had persistent TB symptoms at two months of follow-up, but only 3% remained symptomatic at six months (see Figure 3). The prevalence of persistent symptoms was similar between possible and confirmed TB cases, with 14% of possible and 18% of confirmed cases having symptoms at two months (p = 0.121), and 1% of possible and 0% of confirmed TB cases having TB symptoms at six months follow-up (p = 0.072). TB treatment outcomes between Xpert-positive and negative TB cases were not different (p 0.460). Among the 48 Xpert-positive cases started on treatment, 48% had a successful treatment outcome (six month treatment completion or cure), 25% transferred out, 23% defaulted, 2% were still on treatment and 2% died. Among the 58 Xpert-negative patients started on treatment, 64% completed or cured, 19% transferred, 12% defaulted, 3% still on treatment, and 2% died. Among TB suspects classified as not having TB, 1% died (3/535). TB treatment outcomes were also similar between confirmed and possible TB cases (p = 0.262).

During follow-up, a similar proportion of HIV co-infected Xpert positive individuals (55%) started ART as co-infected Xpert negatives (51%, p 0.130). The median time to starting ART was similar among Xpert positives, Xpert negative TB patients and those without TB (23 days [IQR: 16–56], 24 days [IQR: 19–54] and 22 days [IQR: 14–31] respectively).

## Discussion

In this prospective cohort of TB suspects with high HIV prevalence screened by Xpert at point-of-care, 8% tested positive on initial Xpert and an additional 4% were bacteriologically confirmed with TB on culture, smear or second Xpert. Surprisingly less than half of those initiated on TB treatment had a positive Xpert result on initial screening, and 41% initiated treatment based on clinical or radiological findings alone.

Those diagnosed with TB were more likely to be HIV-infected, ART-naive, have a lower CD4 count and present with more symptoms, as could be expected in a setting with high HIV prevalence [16]. Xpert-negative, culture-positive TB cases had a lower sputum bacillary burden, with significantly longer time to culture positivity compared to Xpert-positives. Xpert-negative TB cases were similar to Xpert-positive cases on most clinical characteristics, including HIV status, CD4 count and ART use. This is in contrast to findings from Cape Town, where Xpert-negative TB cases had less advanced immunosuppression compared to Xpert-positive cases [13]. Differences in the study populations may partially explain the discrepancy. Our study population was more heterogeneous in terms of HIV infection, level of immunosuppression and ART experience, and thus more generalizable to the target population for Xpert testing according to the South African implementation strategy than the population studied by Lawn et al, which was restricted to ART-eligible HIV-infected persons regardless of TB symptoms.

Point-of-care Xpert did afford a clear advantage to patients in terms of time to TB treatment initiation. Almost all TB suspects testing Xpert-positive were started on TB treatment the same day as presenting with TB symptoms. This was two weeks faster than Xpert-negative suspects who started empirically or based on suggestive chest x-ray, and 20 weeks faster than those diagnosed by culture. Only 70% of those diagnosed based on culture were ever started on treatment, as some patients did not return to the clinic following the long delay for results, which is consistent with findings from other clinics in South Africa [17]. Furthermore, 12% of all culture results were either contaminated, not reported, or otherwise missing, highlighting the limited clinical value of culture. Despite this faster TB treatment initiation, Xpert-negatives had similar six-month TB treatment outcomes, including mortality, confirming findings by Lawn et al [13] and Yoon et al [18], and time to antiretroviral treatment initiation.

Xpert-negative TB cases who started treatment based on clinical or radiological criteria only were more likely to be HIV-infected, but were otherwise clinically similar to bacteriologically confirmed cases. This likely reflects a clinician’s decision to initiate TB treatment in the absence of a positive confirmatory diagnostic in HIV-infected individuals to avoid the high mortality of untreated TB in this population [19]. The finding that more than half of all patients starting TB treatment were Xpert-negative, and 41% started TB treatment based on radiological or clinical findings was surprising given the high sensitivity of Xpert in this population. These results are similar to a cohort of TB suspects screened by sputum microscopy, in which 44% of TB cases at an HIV clinic in South Africa started treatment based on a suggestive chest x-ray [20]. These findings emphasize the need for evidence-based assessment strategies for Xpert-negative TB suspects.

Our findings should be interpreted taking several limitations into account. Firstly, we studied a single primary care center with a clinical decision making process and access to diagnostic testing that may not be representative of all clinics in South Africa or other regions. Secondly, the observed time to treatment and proportion of patients started on treatment likely represents a “best case” scenario and may not be generalizable to settings where Xpert is performed at centralized laboratories. Thirdly, even though cultures were negative in almost all patients started on treatment based on clinical or radiological suspicion, we cannot exclude the presence of sputum culture-negative TB in these individuals, and that some patients started on treatment may not have had TB. Finally, our assessment of the impact of Xpert at point-of-care on treatment outcomes was limited by small sample size and relatively high (15%) loss to follow-up.

### Conclusions

This operational research, the first to assess the operational impact of the 2010 recommendation for use of Xpert as the initial diagnostic highlights the diagnostic advantage of Xpert at point-of-care as this results in same day treatment initiation, the low mortality among Xpert-negative TB suspects, and the inadequacy of culture and x-ray in terms of speed and accuracy, respectively, for the assessment of Xpert-negative TB suspects. The prevalence of treatment without bacteriological confirmation reflects the complexity of TB diagnosis, even in the Xpert era. Future research should focus on optimizing assessment strategies of Xpert-negative TB suspects, accounting for setting specific factors such as HIV co-infection, laboratory capacity and cost concerns, to ensure the ultimate goal of improved patient outcomes through rapid and accurate diagnosis in all TB suspects.
